# Deep Learning in Radiology: Does One Size Fit All?

**DOI:** 10.1016/j.jacr.2017.12.027

**Published:** 2018-01-31

**Authors:** Bradley J. Erickson, Panagiotis Korfiatis, Timothy L. Kline, Zeynettin Akkus, Kenneth Philbrick, Alexander D. Weston

**Affiliations:** Radiology Informatics Laboratory, Department of Radiology, Mayo Clinic, Rochester, Minnesota

**Keywords:** Deep learning, machine learning, computer-aided diagnosis

## Abstract

Deep learning (DL) is a popular method that is used to perform many important tasks in radiology and medical imaging. Some forms of DL are able to accurately segment organs (essentially, trace the boundaries, enabling volume measurements or calculation of other properties). Other DL networks are able to predict important properties from regions of an image—for instance, whether something is malignant, molecular markers for tissue in a region, even prognostic markers. DL is easier to train than traditional machine learning methods, but requires more data and much more care in analyzing results. It will automatically find the features of importance, but understanding what those features are can be a challenge. This article describes the basic concepts of DL systems and some of the traps that exist in building DL systems and how to identify those traps.

Traditional machine learning identifies patterns that are present in training sets. In those traditional approaches, it is necessary to compute “features” that are thought to be important factors, which are then used as input to train the system how to classify images as positive or negative. The meaning of positive and negative, as well as the features selected, depends on the type of image (eg, CT, MR, PET) as well as the task (eg, determine if cancer is present, or if the lesion is benign versus malignant).

An early form of machine learning was the artificial neural network (ANN) that was based on the human brain. It had inputs (each input was called a node) with multiple connections from one layer of nodes to the next, just as neurons have dendrites that have multiple inputs and pass their signal on to the next neuron, finally outputting to a muscle. However, ANNs did not work very well and fell into disuse.

Deep learning (DL) is a new form of machine learning that has dramatically improved performance of machine learning tasks. DL is interesting not only because its level of performance is greater, but also because it does not require a human to identify and compute the critical features. Instead, during training, DL algorithms “learn” discriminatory features that best predict the outcomes. This means that the amount of human effort required to train DL systems is less (because no feature engineering or computation is required) and may also lead to the discovery of important new features that were not anticipated.

## IMPORTANT DRIVERS OF DL

Moore’s law states that the performance of computing technology doubles about every 18 months. The world of DL has seen a much faster advance in computing speed. The main reason is that graphics processing units (GPUs) used for displaying graphics on computer screens can also be used to perform DL calculations. GPUs typically have hundreds or thousands of processing units that perform multiple calculations simultaneously. It is therefore typical to see DL algorithms run 10 to 100 times faster when GPUs are used compared with traditional CPUs. Another important factor is that the memory on the GPU cards is increasing rapidly, which is critical for DL applications.

Billions of dollars have been invested in DL technologies across their scope of application, which includes Internet search, social media, and self-driving vehicles. Recent versions of GPUs now have special adaptations to further improve DL performance, and special computers with multiple GPUs designed specifically for DL have been built and sold. Companies are now building special purpose processors that can perform the DL computations even more efficiently than GPUs. These devices are focused both on being efficient in the training of the models as well as inference (making the prediction after being trained).

Although the rapid advance of technology is often cited as *the* reason for DL success, advances in algorithms are probably more responsible for the recent success. These advances addressed the problems seen in ANNs. DL networks get their name because they have many layers; most systems now have 30 to 150 layers, compared with traditional ANNs that would fail if they had more than about 3 layers.

One important advance that enabled DL is improved activation functions. An activation function converts the weighted sum of inputs into an output value that is then conveyed to nodes in the next layer. Activation functions add nonlinearity, which is a critical element of learning. Early neural networks used sigmoidal activation functions because neurons had sigmoidal activation functions, but they are susceptible to phenomena that can halt network learning or lead to network instabilities. When there are millions of nodes that can be altered, it is critical to identify those nodes that can produce the greatest improvement. In some cases, it can be hard to identify which ones to alter, a situation known as the vanishing gradient problem.

**Fig 1:**
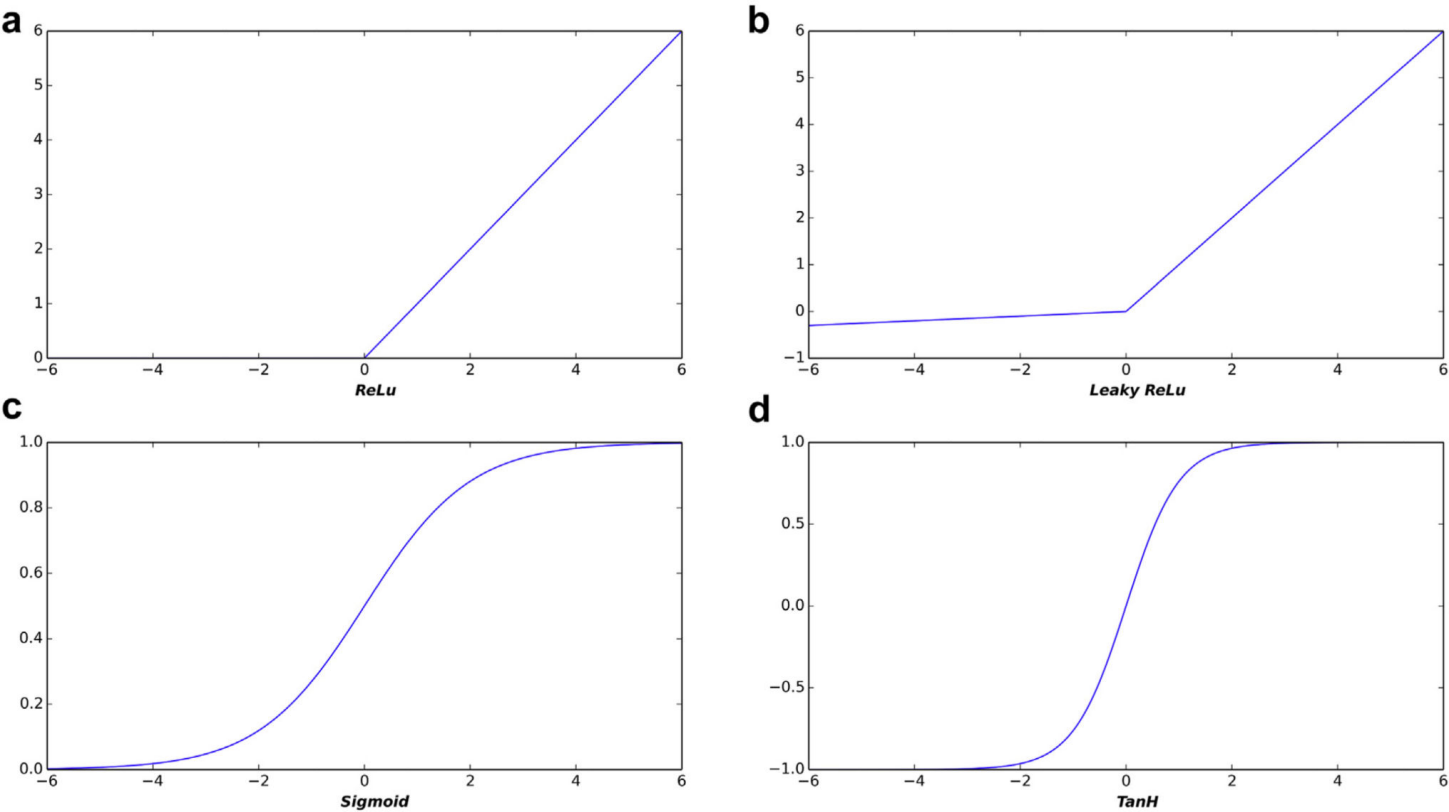
Example of three activation functions used in neural networks: (a) rectified linear unit (ReLU), (b) leaky ReLU, (c) sigmoid, and (d) Tanh. Traditional neural networks used sigmoidal functions that simulated actual neurons, but are less effective in current networks, likely because they do not adequately reward very strong activations.

For DL systems, other simpler activation functions, such as rectified linear unit (ReLU), have been proven to work better than sigmoidal functions. The ReLU takes the weighted sum of inputs, and, if that is less than zero, it outputs a zero; otherwise, it outputs the input [1–3]. More recent variants (eg, leaky ReLU [4]) produce small negative values for negative inputs but give the same output as input when positive. Figure 1 shows sigmoidal, ReLU, and leaky ReLU activation functions. These activation functions are actually much simpler to compute than sigmoidal functions—evidence that computing power is not the only factor in DL success.

Overfitting poses another challenge to training deep neural networks. During training, networks can learn the specific examples in the training set that are not broadly predictive features for the problem of interest if they have enough parameters compared with the number of examples. To paraphrase Einstein, we want the simplest (fewest layers or nodes) that solves the problem, but no simpler. The simpler the network, the less it will be able to learn the specifics of the training set. For instance, suppose we wish to train a network to classify liver masses as benign or malignant. If we have a small set of training CT scans, we might have a situation where most of the malignant masses are found in men and most of the benign are found in women. Also suppose that we had a very powerful (many parameters) network that can identify all the organs in the abdomen and pelvis. In that case, the algorithm might detect the prostate as a strong predictor of malignancy, but this will not likely generalize well to the larger population.

This is why it is critical to have a “holdout” test set to test the trained system. Overfitting can often be identified when a model begins to perform significantly worse on testing data than on the training data.

Dropout regularization is another simple technique used to reduce overfitting [1]. Dropout regularization works by randomly removing a subset of the network nodes during each training epoch, which changes the composition of the network across training epochs. As a result, the model generates predictions that are less reliant on specific neurons in the network, which reduces the chance the network will make spurious associations. Other techniques to address overfitting boost the size and variability of the training set and include batch normalization and data augmentation, utilizing loss weightings to amplify the loss on categories that are underrepresented in the training set.

## COMPONENTS OF A DL SYSTEM—LAYERS AND ARCHITECTURES

Compared with a traditional neural network where all nodes of a layer are connected to those of subsequent layers (“fully connected”), modern DL systems typically consist of several specialized layers. Fully connected layers usually are just the final layers that combine the learned features to make decisions. When images are the input, it is typical to use convolutions as input layers, and that is the basis for their name “convolutional neural networks” (CNNs). CNNs were proposed many years ago but were not effective until some of the computational and algorithmic solutions noted above were achieved. The size of the input convolutional kernel is defined by the scientist as part of the network architecture, but the values of the kernel itself are learned by the system.

**Fig 2:**
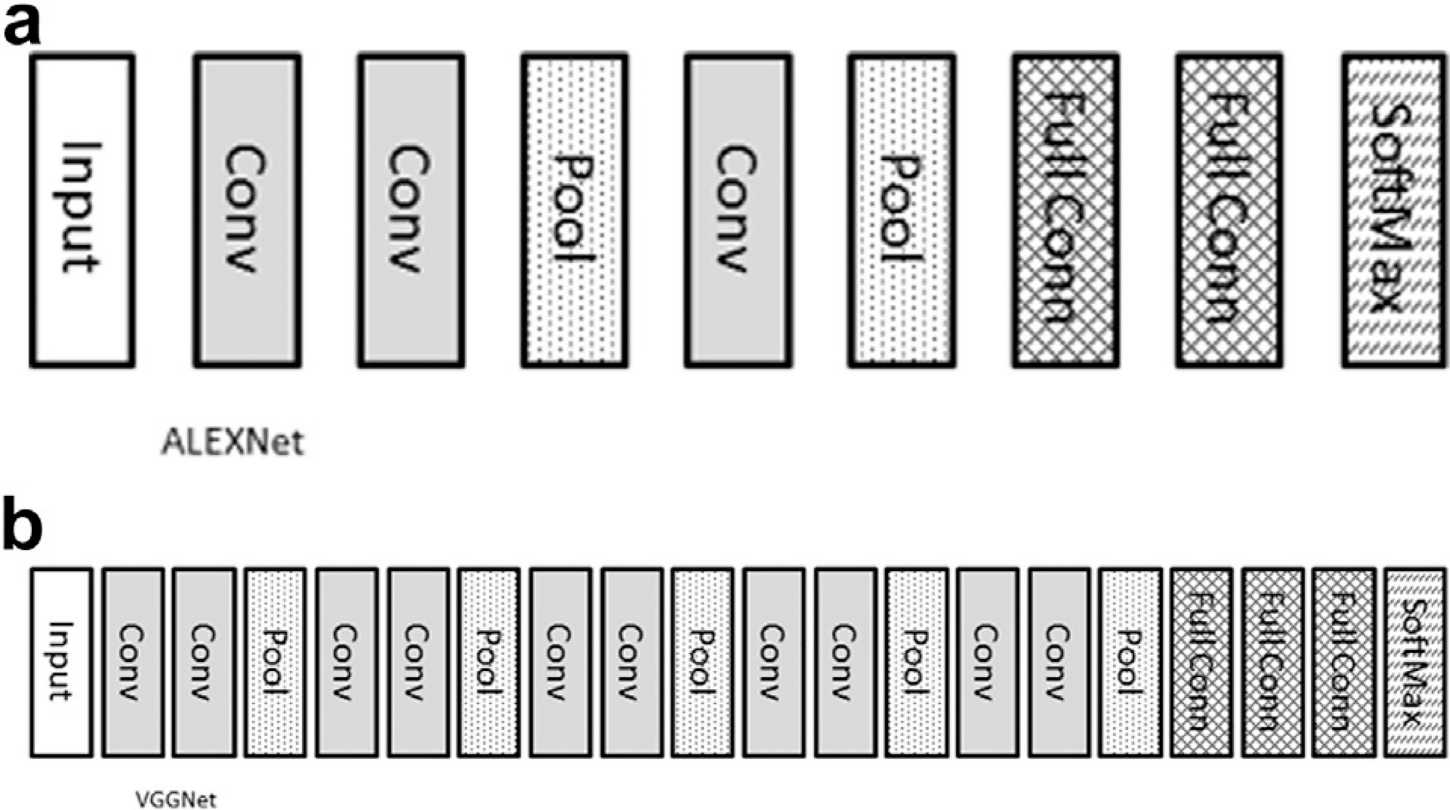
Architecture of two popular networks: (a) AlexNet and (b) VGGNet. Input, input image; Conv, convolutional layer; Pool, maximum value pooling layer; Full Conn, fully connected layer; SoftMax, softmax function, also known as normalized exponential function that takes an input vector and maps it to the range of (0,1). This is the class probability. If there are 1,000 outputs in this layer, each value in the 1,000-element vector would correspond with the probability of the input image being that class. The highest value(s) are the predicted class(es).

In many cases, one or two convolutional layers will be followed by a pooling layer. A popular pooling function is to take the maximum value of inputs, known as max pooling. Max pooling thus takes the maximum value of the convolutional layer for the region of the image. In this way, max pooling layers identify the most predictive feature within the sampled region and reduce the resolution and memory requirements of the image. It is common to have several groups of convolution and pooling. It has been shown that having several 3 × 3 kernels gives as good or better results and is computationally more efficient than a few larger kernels [5]. Recently, special instructions to perform 3 × 3 convolutions in hardware have further increased their speed and advantages. Figure 2 shows the architecture of two popular networks: AlexNet [6] and VGGNet [5].

After repeated application of convolution and pooling, the output of the last pooling layer is usually fed into several fully connected network layers. It is within these layers that the predictive weights are used to translate the output of the final pooling layer into the output. Newer forms of CNNs, such as network in network and inception, have demonstrated that terminal fully connected layers may not be necessary and instead may be computed directly from the terminal convolution or pooling layer [7,8].

These are the specific layers in a DL system. They are the building blocks used to construct a complete network. There are many options for how these building blocks are arranged to achieve certain goals. Original designs (eg, VGG16) were fairly simple linear designs starting with convolutions and ending with fully connected networks. More recently, residual network layers have been applied [9], in which the output of a layer is forced to learn something by using a parallel connection between layers. This “competition” between layers has been shown to significantly increase performance [10].

It is possible to connect the layers in even more complex ways. For instance, one might have significantly different convolution kernels fed into their own separate parallel layers, with the output of those combined to a final output network. This can allow one part (those with small convolutional kernels) of the network to focus on learning small or high-resolution features and another part (large kernels) to focus on global features. One recent report [11] used this approach for brain tumor segmentation with good results.

Another option is to stack the networks to perform sequential tasks. In this case, the first group of layers might perform segmentation, such as by finding the liver. The second group of layers might then identify lesions within the liver. One recent report [12] on liver lesion segmentation showed good results, another has used it for finding microbleeds on head CTs [13], and we have recently published using this approach in which T2 images of the head are provided as input and the genomic properties of the tumor in the brain are computed.

## HOW MUCH DATA IS ENOUGH?

DL and big data are often confused, because it is thought that any DL system requires huge amounts of data. Certainly, early versions of photographic recognition systems like ImageNet did use large training sets. However, it is less clear that this requirement exists for medical images. Recognition of objects like cats and dogs in photographs has to deal with significant variations in appearance due to lighting, geometry (photo being taken from front, or side, or top, or back; close or far; with another object partially occluding it), and variations in appearance (brown dogs, black dogs, yellow dogs, spotted dogs). Medical images have well-known scale, and the variation in appearance of normal structures can be small compared with the variations described above, depending on the specific problem. Therefore, the amount of data really depends on the variability seen in the process being studied, but huge datasets are not always required.

One way to reduce the need for training data is to train the first layers of the system on other images that are expected to have similar features that are important—a technique referred to as transfer learning. Although this might sound restrictive, there has been great success in using networks trained on photographic images and then training the last few layers to make radiological diagnoses [14,15]. Whether there is significant room for improvement by starting with network trained on medical images is not clear and remains an area of active investigation.

Another way to reduce the need for training data is to create variants of the original data—a technique called data augmentation [16]. For instance, one might create “new” images by rotating the original image by 1, 2, 3, or more degrees clockwise or counterclockwise or by flipping the image top to bottom or left to right. Adding noise to the image is another data augmentation technique. These variants of the original image can keep the DL system from learning the specific training examples. We and others have shown this to be quite effective for medical images.

## ADAPTING DL MODELS TO BEST ADDRESS A CLASS OF PROBLEMS

Unfortunately, there is currently no rule of thumb for selecting the appropriate CNN architecture for a given image classification task. Numerous network architectures have been developed for general purpose image classification (eg, VGG16, Inception, ResNets). These networks have been typically designed to perform image classification on very large and diverse datasets (eg, ImageNet). To give good results, general purpose classifiers are typically capable of learning an enormous set of image features and as a result are compute-intensive to train. However, bigger is not always better. For smaller and possibly more homogenous medical imaging datasets, more complex general purpose classifiers may actually perform poorer than simpler models. Large general purpose classifiers are prone to rapidly overfit small training datasets and as a result can fail to learn the discriminatory features contained in a small dataset. In contrast, smaller classifiers capable of capturing fewer discriminatory features may perform better because they are conceptually forced to learn more generic features. However, many of the architectural innovations pioneered in large general-purpose classifiers (eg, residual blocks, inception blocks) do not directly dictate model size (eg, number or size of the CNN layers) and likely have applicability in classifiers designed for medical imaging. At present, determining the applicability of these architectural innovations to a dataset classification problem requires experimentation.

In general, the depth of a network is related to the image size, but the selection of precise network architecture has to be studied before one can be assured that a reasonably optimal architecture is being used. A good solution for parameter optimization is offered by grid search techniques. If one uses transfer learning, the architecture of the network generally needs to be similar to the network of the original system that the weights came from.

The type of problem being addressed also impacts the architecture. For image segmentation, the most commonly utilized architectures are the fully convolutional networks [17], autoencoders [18], and UNETs [19]. These techniques have been successfully applied to segmentation of several types of medical images, including brain [20,21], lung [22], prostate [23], and kidney [24]. For image classification, CNNs have been the most common architecture.

The traditional view of machine learning and neural networks is that a given system can only solve one well-defined problem, which is known as narrow artificial intelligence (AI). The ability to address any problem that might be presented to a system is known as general artificial intelligence. Building such a general system seems critical to medical image interpretation because it is rare for an examination to have only one question (mammography is close: in nearly all cases, the question is breast cancer or not, with breast density perhaps being a second question).

Although most publications have presented narrow AI solutions, one recent publication [25] has shown that it is possible to have one network address a range of problems and that adding this breadth does not degrade performance compared with any individual narrow AI solution.

## WHAT DOES A DL SYSTEM “SEE”?

An important challenge to clinical adoption of DL systems is that because DL does not start with a human selecting features that will be evaluated, knowing the features that are actually used for interpretation seems impossible. Some feel that if the basis for a decision is not known, DL cannot be valid or relied upon for medical care. Placing features into an image, or removing them, and then assessing performance, is one approach that has been described for revealing the important factors in classification by a trained CNN [26]. In this case, if hiding some part of anatomy or feature like edges results in degraded performance of a trained network, one can conclude that those features are important.

Alternatively, the creation of saliency maps [27] and attention maps [28] have been suggested as a method to visualize relative importance of various regions of an image to a classifier’s classification. These techniques can be used to generate heat map–like images that categorize the relative importance to pixels or voxels within an image to a classifier’s categorization. This has also been applied to nonradiological images like dermatology images, in which saliency maps of skin cancer images were generated that revealed the pixels that most influence a CNN’s prediction [29].

Another approach to understanding DL networks is to convert the weights of the fully connected network into a more familiar form. Recent reports [30,31] showed the conversion of network weights into decision trees, essentially a series of if-then decisions based on criteria used by the network. Although there was slight loss in overall accuracy, this approach can give a strong sense of the information that is important to making decisions.

Whether the previously discussed approaches will fully capture all the features and weights in a way that can be understood by a human is unclear, but it is certainly likely that at least some unexpected new features will be found —this is a critical element of discovery science. Although much radiology in the past has been hypothesis-driven, discovery science is needed to help us make the next major steps forward by identifying unexpected or otherwise unobserved findings in our data. Tools that can highlight these findings are essential to the vitality of our specialty. This is one reason why DL seems to be a critical tool for advancing radiology.

## CONCLUSION

DL is at once both the natural evolution of prior technologies accelerated by improvements in algorithms and computing power and a dramatic leap forward in our ability to extract critical information in images that may be difficult to observe with standard techniques. We are still in the early stages of applying DL, and building DL systems is much more an art than a science when deciding the optimal architecture of a DL network for a particular problem.

## TAKE-HOME POINTS

■ DL has dramatically improved the performance of computer algorithms outside of medicine, and it can be expected to dramatically improve performance of medical applications in the near future.
■ There continue to be important advances in theory around DL, including both improved learning techniques and better understanding of optimal architectures for certain classes of problems.
■ Although we may not “see” what DL applications “see” in many cases, there are ways to better understand the important features used by DL algorithms that will provide insight into their operation.
